# Association between increased C-reactive protein and cardiovascular disease among patients with rectal cancer

**DOI:** 10.3389/fonc.2023.1287619

**Published:** 2023-11-22

**Authors:** Huimin Qiao, Changxin Wang, Chunhong Yang, Lei Lei, Yijing Chen, Yun Luo, Xiangfu Zeng, You Guo

**Affiliations:** ^1^ Medical Big Data and Bioinformatics Research Centre, First Affiliated Hospital of Gannan Medical University, Ganzhou, China; ^2^ School of Public Health and Health Management, Gannan Medical University, Ganzhou, China; ^3^ Department of Gastrointestinal Surgery, First Affiliated Hospital of Gannan Medical University, Ganzhou, China; ^4^ Studies Section, First Affiliated Hospital of Gannan Medical University, Ganzhou, China; ^5^ Department of Cardiology, Luotian County People’s Hospital, Huanggang, China

**Keywords:** C-reactive protein, rectal cancer, cardiovascular disease, chemotherapy, inflammation

## Abstract

**Purpose:**

This study aimed to investigate the association between increased C-reactive protein (CRP) and cardiovascular disease (CVD) in individuals with rectal cancer, as well as to understand the effect of chemotherapy for cancer on increasing CRP and its underlying mechanisms.

**Patients and methods:**

From January 1, 2010 to December 31, 2020, individuals with rectal cancer were evaluated at the First Affiliated Hospital of Gannan Medical University. Then, in patients with rectal cancer, the relationship between increased CRP and CVD attributes was summarized, and the impact of chemotherapy on CRP levels was qualitatively assessed. For further investigation into potential regulatory mechanisms of CRP, differentially expressed genes (DEGs), GO and KEGG enrichment analyses were conducted.

**Results:**

A total of 827 individuals were included in the study, including 175 with CVD (21.16%) and 652 without CVD. A significant association between increased CRP and CVD events was observed in rectal cancer patients (p < 0.01), and it significantly improved the classification performance of the CVD predictive model in the AUC (0.724 vs 0.707) and NRI (0.069, 95% CI 0.05-0.14). Furthermore, a comparison of CRP levels before and after chemotherapy revealed a significant increase among rectal cancers post-treatment (p < 0.001). Analysis of differentially expressed genes and co-expression indicated that 96 DEGs were involved in the pathophysiology of increased CRP after chemotherapy, and three hub genes were implicated in atherosclerotic susceptibility.

**Conclusion:**

In conclusion, our findings indicated that increased CRP levels following chemotherapy profoundly impacted CVD events in individuals with rectal cancer, and may be beneficial in promoting CVD prediction in clinical practice.

## 1 Introduction

Multidisciplinary management has significantly extended the survival of patients with rectal cancer, increasing the number of rectal cancer survivors (1–3). Subsequently, the problem of chronic cardiovascular disease is also highlighted in these survivors, who are more predisposed to developing cardiovascular disease (CVD) than those without a history of cancer (4, 5). More importantly, developed CVD drastically reduces survival and primarily contributes to non-rectal deaths (6, 7). It follows that uncovering the characteristics and mechanisms of CVD in patients with rectal cancer has become a current focus of research.

Among individuals, inflammation promotes the development of CVD (8, 9). C-reactive protein (CRP) is an acute-phase protein produced primarily in the liver, which has been successfully regarded as an indicator of inflammation (10, 11). CRP is currently a critical indicator in assessing residual inflammation in patients with CVD (12), and elevated CRP is an independent predictor of cardiovascular event recurrence and mortality (13). However, in individuals with rectal cancer, no data are available verifying the association between CRP and CVD.

Chemotherapy is the most commonly accepted treatment for rectal cancer. Frequently used chemotherapy can damage endothelial cells, contributing to endothelial apoptosis, loss of vascular integrity, severe vascular dysfunction, and thrombus formation (14–16). Alternatively, some cytotoxic chemotherapy drugs can also trigger inflammatory signaling cascades in endothelial cells (17, 18). But it is unknown whether chemotherapy causes elevated CRP levels and promotes the development of CVD via CRP.

In this study, we conducted a retrospective study to investigate the association between CRP and CVD in patients with rectal cancer. We further analyzed differences in levels of CRP among rectal cancer patients to evaluate the impact of chemotherapy on CRP. Furthermore, we explored the role of the CRP gene in chemotherapy and the vascular endothelium using gene expression profile datasets.

## 2 Materials and methods

### 2.1 Study population

**Figure 1:**
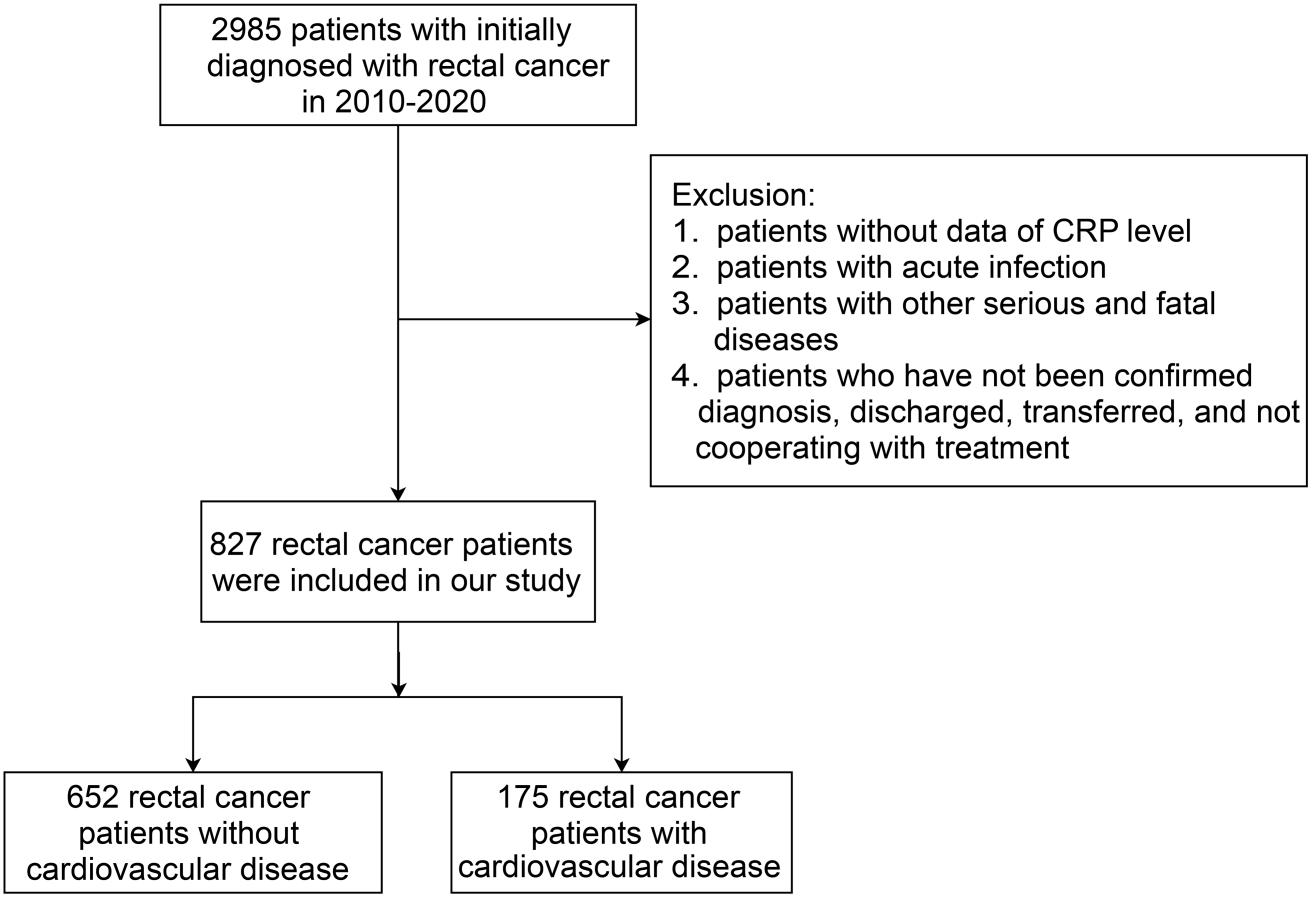
The flow chart of the study population recruitment.

The study retrospectively collected data of rectal cancer patients with no prior history of CVD, who were initially diagnosed from January 1, 2010 to December 31, 2020, in the First Affiliated Hospital of Gannan Medical University. The composite primary outcome was the incidence of one or more CVDs after treatment for rectal cancer patients during inpatient hospital visits. The cardiovascular complications of rectal cancer treatment can be divided into nine categories (19): 1) Coronary artery disease; 2) Arrhythmia; 3) Congestive heart failure; 4) Valvular disease; 5) Pulmonary hypertension; 6) Thrombotic disease; 7) Peripheral vascular disease; 8) Stroke; 9) Pericardial complication. According to the tenth International Classification of Diseases (ICD-10 codes), patients with a first-time diagnosis of rectal cancer (C19-C20) and with CRP measurement data were eligible. The patients were excluded according to the following criteria: 1) Missing CRP data; 2) Occurrence of an acute infection; 3) Having other serious and fatal diseases; 4) Those who did not confirm the diagnosis, were discharged, transferred, and uncooperative with the treatment. The flow chart of the study population recruitment is shown in **Figure 1**.

From January 1, 2015 to December 31, 2020, data on patients with CVD was collected from the First Affiliated Hospital of Gannan Medical University. A total of 175 patients with CVD were identified through 1:1 matching on gender, age, and nine categories of CVD of rectal cancer patients with CVD.

The following indicators were extracted from the electronic medical record: age, gender, CRP, chemotherapy, neutrophils, lymphocytes, white blood cell (WBC) count, eosinophils, basophils, carcinoembryonic antigen (CEA), hyperglycemia, blood lipids, an infection history, and surgical therapy. In the laboratory, serum CRP is determined by means of transmission turbidimetry. For rectal cancer patients treated with chemotherapy, the records of their serum CRP levels were extracted more than two weeks after the chemotherapy. For the indicators of blood analytes, the patients were categorized as normal and abnormal according to laboratory results (including neutrophils, lymphocytes, WBCs, eosinophils, basophils, and blood lipids). Hyperglycemia was defined as a whole blood glucose level greater than 150mg/dl or 8.5mmol/l without diabetes (20); Dyslipidemia was defined as one of the following conditions according to the 2016 Chinese Adult Dyslipidemia Prevention Guidelines (21): total cholesterol ≥ 6.2mmol/L; triglyceride ≥ 2.3mmol/L; low-density lipoprotein cholesterol  ≥4.1mmol/L; and high-density lipoprotein cholesterol < 1.0mmol/L. The data extracted in this study were only relevant medical records obtained from patients and did not involve personally identifiable information, which met the ethical review requirements.

### 2.2 Statistical analysis

CRP, a continuous variable, was logarithmically transformed. Then the intersection of the two normal curves of the two groups (with and without CVD in rectal cancer patients) was used to determine the cut-off value of CRP (3.3 mg/L). Categorical variables were expressed as frequencies (percentages) and were compared for intergroup differences using the χ^2^ test. Normally distributed continuous variables between two groups were compared using a t-test. Otherwise, the Kruskal-Wallis test was applied. The multivariable logistic regression model was used to identify the independent effects of CRP on CVD in patients with rectal cancer using the significant variables (p<0.05) in the univariable logistic regression analysis. The matching of the propensity score was performed in a 1:1 ratio to adjust for confounding factors and further determine the independence of CRP. The discrimination capability of the prediction model was assessed by examining the change in the area under the receiver operator characteristic curve (ROC AUC) and net reclassification improvement (NRI) when CRP was included in multivariable models. Multiple logistic regression models were built to assess the association between the risk of CRP and coronary artery disease. Subgroup analysis based on gender was used to estimate a specific association between CRP and CVD. In all regressions, odds ratios (ORs) and their 95% confidence intervals (95% CIs) were calculated. All analyses were performed using the R 4.2.2 software. The ggplot2 package was used to make the violin and box plots. Statistical significance was considered as a two-sided p < 0.05.

### 2.3 Bioinformatics analysis

To gain a better understanding of the mechanism that determines CRP expression levels based on the genotype, we retrieved gene expression profile datasets from the Gene Expression Omnibus (GEO) database (http://www.ncbi.nlm.nih.gov/geo/). The R limma package was then used to identify differentially expressed genes, and ClusterProfiler of the R package was employed for Gene Ontology (GO) analysis. Moreover, the Kyoto Encyclopedia of Genes and Genomes (KEGG) Pathway gene annotation was obtained from the Database for Annotation, Visualization, and Integrated Discovery (DAVID) tool (https://david.ncifcrf.gov/home.jsp). All functions with a p-value of less than 0.05 were considered statistically significant.

GSE32384 (22) had 24 liver tissue samples from colorectal liver metastases (CRLM), including 19 samples with oxaliplatin-based chemotherapy and 5 samples without any chemotherapy. We first assessed the difference in CRP levels between the chemotherapy and the non-chemotherapy groups. Subsequently, we conducted DEGs analysis between two groups (FDR <0.1). Functional enrichment analysis (GO biological process) was conducted on the identified DEGs to discover biological functions involved in the CRP gene.

GSE20060 (23) had 385 samples (including replicates of the same sample) from human aortic endothelial cells treated with or without oxidized phospholipids. After averaging the expressed values of the same samples, a total of 96 control and 96 treatment samples were obtained. Then DEGs were calculated between the two groups (FDR <0.01) and the CRP-related genes from DEGs were identified. GO and KEGG pathway analyses were used for enrichment analysis.

We also identified hub genes by selecting common DEGs that were significant in both datasets, thus providing an additional list of gene functions.

## 3 Results

### 3.1 The difference levels of CRP in patients

**Figure 2:**
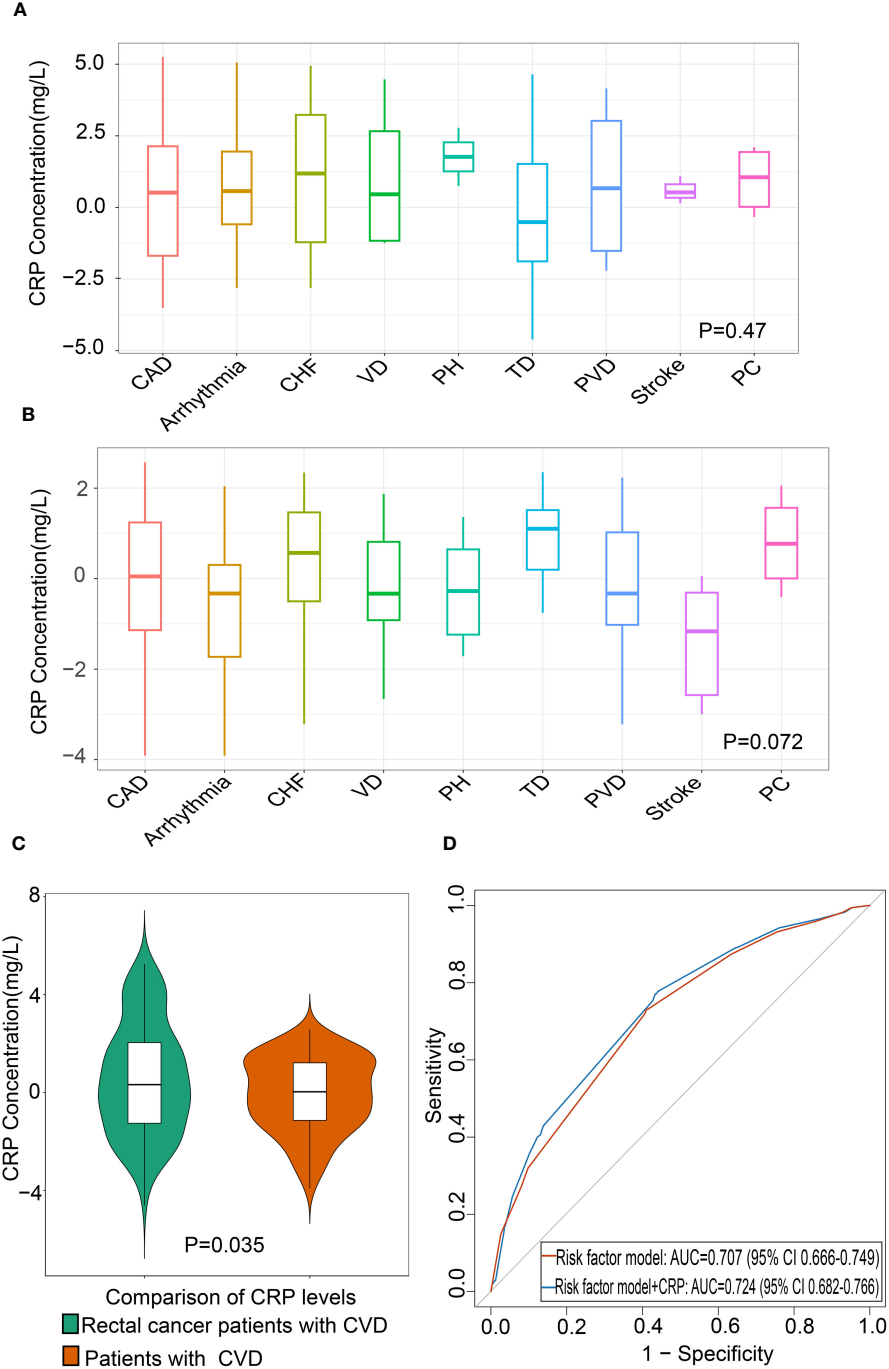
Association between CRP and CVD in patients with rectal cancer **(A)** Differential levels of CRP in nine categories of CVD in patients with CVD (n=175). **(B)** Differential levels of CRP in nine categories of CVD in rectal cancer patients with CVD (n=175). **(C)** Comparison of CRP levels between rectal cancer patients with CVD and patients with CVD. **(D)** The area under the receiver operating characteristic curve of CRP of CVD in rectal cancer patients. The red line indicates the base risk factor model including age, carcinoembryonic antigen (CEA), infection history, and chemotherapy, and blue line indicates the addition of CRP to the base risk factor model. CAD, Coronary Artery Disease; CHF, Congestive Heart Failure; VD, Valvular Disease; PH, Pulmonary Hypertension; TD, Thrombotic Disease; PVD, Peripheral Vascular Disease; PC, Pericardial Complication; CRP, C-reactive protein; CVD, Cardiovascular disease.

A total of 2,985 patients with rectal cancer were diagnosed from 2010 to 2020, of whom 827 met the mentioned exclusion criteria and were selected for our study, including 175 with CVD and 652 without CVD following treatment. The clinical characteristics of the two groups were represented in **Table 1**. Afterwards, we analyzed CRP levels among nine categories of CVD in rectal cancer patients with CVD and in patients with CVD, respectively. The nine categories of CVD among these patients were shown in **Table 2**. The results showed that there were no differences in CRP levels among the nine categories in the two populations (**Figures 2A, B**), indicating a sensible combination of these categories for CVD.

**Table 1:** Clinical characteristics of rectal cancer patients with or without CVD.

Variable	Without CVD Number (%)	With CVD Number (%)	*P*-Value
Total	652 (78.84)	175 (21.16)	
Age, year			<0.001
≤50	124 (19.02)	13 (7.43)	
≥51	528 (80.98)	162 (92.57)	
Gender			0.5
Female	215 (32.98)	53 (30.29)	
Male	437 (67.02)	122 (69.71)	
CRP, mg/L			<0.001
≤3.3	610 (93.56)	147 (84)	
> 3.3	42 (6.44)	28 (16)	
Chemotherapy			0.088
No	239 (36.66)	52 (29.71)	
Yes	413 (63.34)	123 (70.29)	
Neutrophil			0.44
Normal	340 (52.15)	97 (55.43)	
Abnormal	312 (47.85)	78 (44.57)	
Lymphocyte			0.012
Normal	470 (72.1)	109 (52.29)	
Abnormal	182 (27.9)	66 (37.71)	
WBC count			0.793
Normal	402 (61.66)	106 (60.57)	
Abnormal	250 (38.34)	69 (39.43)	
Eosinophil			0.216
Normal	435 (66.72)	108 (61.71)	
Abnormal	217 (33.28)	67 (38.29)	
Basophil			0.82
Normal	646 (99.08)	173 (98.86)	
Abnormal	6 (0.92)	2 (1.14)	
CEA			0.002
Normal	475 (72.85)	147 (84)	
Abnormal	177 (27.15)	28 (16)	
Hyperglycemia			0.019
No	113 (17.33)	44 (25.14)	
Yes	539 (82.67)	131 (74.86)	
Blood lipids			
Normal	316 (48.47)	79 (45.14)	0.435
Abnormal	336 (51.53)	96 (54.86)	
Infection history			<0.001
No	583 (89.42)	117 (66.86)	
Yes	69 (10.58)	58 (33.14)	
Surgical therapy			0.25
No	300 (46.01)	72 (41.14)	
Yes	352 (53.99)	103 (58.86)	

CVD, cardiovascular disease; CRP, C‐reactive protein; WBC, White Blood Cell; CEA, carcinoembryonic antigen;

**Table 2:** Nine main categories of CVD in rectal cancer patients with CVD and patients with CVD.

Categories	Rectal patients With CVD (n=175)	Patients With CVD (n=175)
Coronary artery disease	40 (22.86)	57 (32.57)
Arrhythmia	59 (33.71)	23 (13.14)
Congestive heart failure	11 (6.29)	38 (21.71)
Valvular disease	4 (2.29)	6 (3.43)
Pulmonary hypertension	2 (1.14)	13 (7.43)
Thrombotic disease	44 (25.14)	7 (4.0)
Peripheral vascular disease	6 (3.43)	21 (12.0)
Stroke	3 (1.71)	6 (3.43)
Pericardial complication	6 (3.43)	4 (2.29)

values in parentheses are percentages

### 3.2 Association between CRP and CVD in patients with rectal cancer

To recognize whether CVD in rectal cancer patients was distinct from that in CVD patients, we compared the CRP levels between the two groups. The results showed that the CRP levels were significantly higher in rectal cancer patients with CVD than those in patients with CVD (**Figure 2C**). This finding indicated that rectal cancer patients with CVD should not be confused with CVD patients.

To further identify the relationship between CRP and CVD in patients with rectal cancer, we conducted a univariate logistic regression analysis. The results found that increased CRP was significantly associated with CVD in rectal cancer patients (OR: 2.767, 95% CI 1.646-4.592, p < 0.001). Additionally, the other five variables were significant factors in the univariate analysis, as shown in **Table 3**. To control for the potential influence of these variables on CRP, we performed multivariable logistic regression using the significant factors in the univariable regression. The results showed that CRP remained a risk factor for CVD in patients with rectal cancer (OR: 2.411, 95% CI: 1.378-4.165, p=0.002). Furthermore, 1:1 propensity score matching with a caliper value of 0.1 was performed to verify the stability of the above findings. The propensity score matched 332 pairs of rectal cancer patients with and without CVD. The results consistently demonstrated that CRP was considered as an independent risk factor for CVD in patients with rectal cancer (**Table 4**).

**Table 3:** Univariable and multivariable logistic regression analysis of the association between CRP and CVD in patients with rectal cancer.

Variable	Univariable		Multivariable	
	OR (95%CI)	*p*-Value	OR (95%CI)	*p*-Value
Age				
≤50	Reference		Reference	
≥51	2.927 (1.668-5.567)	<0.001	2.940 (1.630-5.726)	0.001
Gender				
Female	Reference		–	–
Male	1.133 (0.793-1.635)	0.5	–	–
CRP, mg/L				
≤3.3	Reference		Reference	–
>3.3	2.767 (1.646-4.592)	<0.001	2.411 (1.378-4.165)	0.002
Chemotherapy				
No	Reference		Reference	
Yes	1.369 (0.959-1.976)	0.089	1.859 (1.264-2.772)	0.002
Neutrophil				
Normal	Reference		–	–
Abnormal	0.876 (0.626-1.224)	0.44	–	–
Lymphocyte				
Normal	Reference		–	–
Abnormal	1.564 (1.098-2.215)	0.012	–	–
WBC count				
Normal	Reference		–	–
Abnormal	1.047 (0.742-1.470)	0.793	–	–
Eosinophil				–
Normal	Reference		–	–
Abnormal	1.244 (0.878-1.753)	0.216	–	–
Basophil				
Normal	Reference		–	–
Abnormal	1.245 (0.181-5.457)	0.79	–	–
CEA				
Normal	Reference		Reference	
Abnormal	0.511 (0.324-0.782)	0.003	0.427 (0.262-0.674)	<0.001
Hyperglycemia				
No	Reference		–	–
Yes	1.602 (1.097-2.371)	0.02	–	–
Blood lipids				
Normal	Reference		–	–
Abnormal	1.143 (0.818-1.600)	0.435	–	–
Infection history				
No	Reference		Reference	
Yes	4.189 (2.800-2.261)	<0.001	4.259 (2.766-6.572)	<0.001
Surgical therapy				
No	Reference		–	–
Yes	1.22 (0.871-1.714)	0.25	–	–

CVD, cardiovascular disease; CRP, C‐reactive protein; WBC, White Blood Cell; CEA, carcinoembryonic antigen;Variables marked with “-” showed no statistical significance in the univariate analysis and were not included in the multivariate analysis.

**Table 4:** Association between CRP and CVD in patients with rectal cancer after propensity score matching.

Variable	OR (95% CI)	*p*-Value
Age		
≤50	Reference	
≥51	0.972 (0.427-2.221)	0.946
CRP		
≤3.3	Reference	
>3.3	4.054 (1.785-10.434)	0.002
Chemotherapy		
No	Reference	
Yes	1.041 (0.645-1.684)	0.869
CEA		
Normal	Reference	
Abnormal	1.035 (0.571-1.876)	0.910
Infection history		
No	Reference	
Yes	1.029 (0.627-1.686)	0.912

CRP, C‐reactive protein; CEA, carcinoembryonic antigen;

To evaluate the value of CRP in the diagnosis of CVD in rectal cancer, ROC AUC and NRI were described. When adding CRP to multivariable models including age, CEA, infection history, and chemotherapy, the ROC AUC showed an increase of 0.17 compared to the model without CRP (**Figure 2D**, **Table 5**). The addition of CRP to risk factor model also improved NRI by 6.9% (95% CI 5–14%). These results demonstrated that CRP improved the accuracy of diagnosing CVD in patients with rectal cancer (**Table 5**).

**Table 5:** Performance metrics of risk factor models with and without CRP.

	Risk factor model^a^	Risk factor model + CRP	*p* -Value
NRI	Reference	0.069 (0.05-0.14)	0.03
Δ AUC	Reference	0.17	0.056

a Risk factor model as the base risk factor model included age, carcinoembryonic antigen (CEA), infection history, and chemotherapy

### 3.3 Association and subgroup analysis

Based on the fact that coronary artery disease (CAD) accounts for the highest proportion of CVD mortality (estimated 1 in 4 deaths) (24, 25), we conducted an investigation into the association between CRP and the risk of CAD. As would be expected, the results showed that CRP was associated with a higher risk of CAD in model 1 (OR: 4.217, 95% CI 1.885-9.434, p**<** 0.001), and in model 2 (OR: 3.531, 95% CI 1.533-8.134, p=0.005) when compared to those non-CAD (**Table 6**).

**Table 6:** Association of CRP with CAD in patients with rectal cancer.

Variable	Without CVD	CAD OR (95% CI)	Non-CAD^a^ OR (95% CI)	*p*-Value
Model 1	1	4.217 (1.885-9.434)	2.379 (1.336-4.237)	<0.001
Model 2	1	3.531 (1.533-8.134)	2.087 (1.122-3.882)	0.005

CVD, cardiovascular disease; CAD, Coronary artery disease;

a Included arrhythmia, congestive heart failure, valvular disease, pulmonary hypertension, thrombotic diseases, peripheral vascular disease, stroke and pericardial complication; Model 1 adjusted for none; Model 2 adjusted for age, chemotherapy, carcinoembryonic antigen (CEA) and infection history

**Figure 3:**
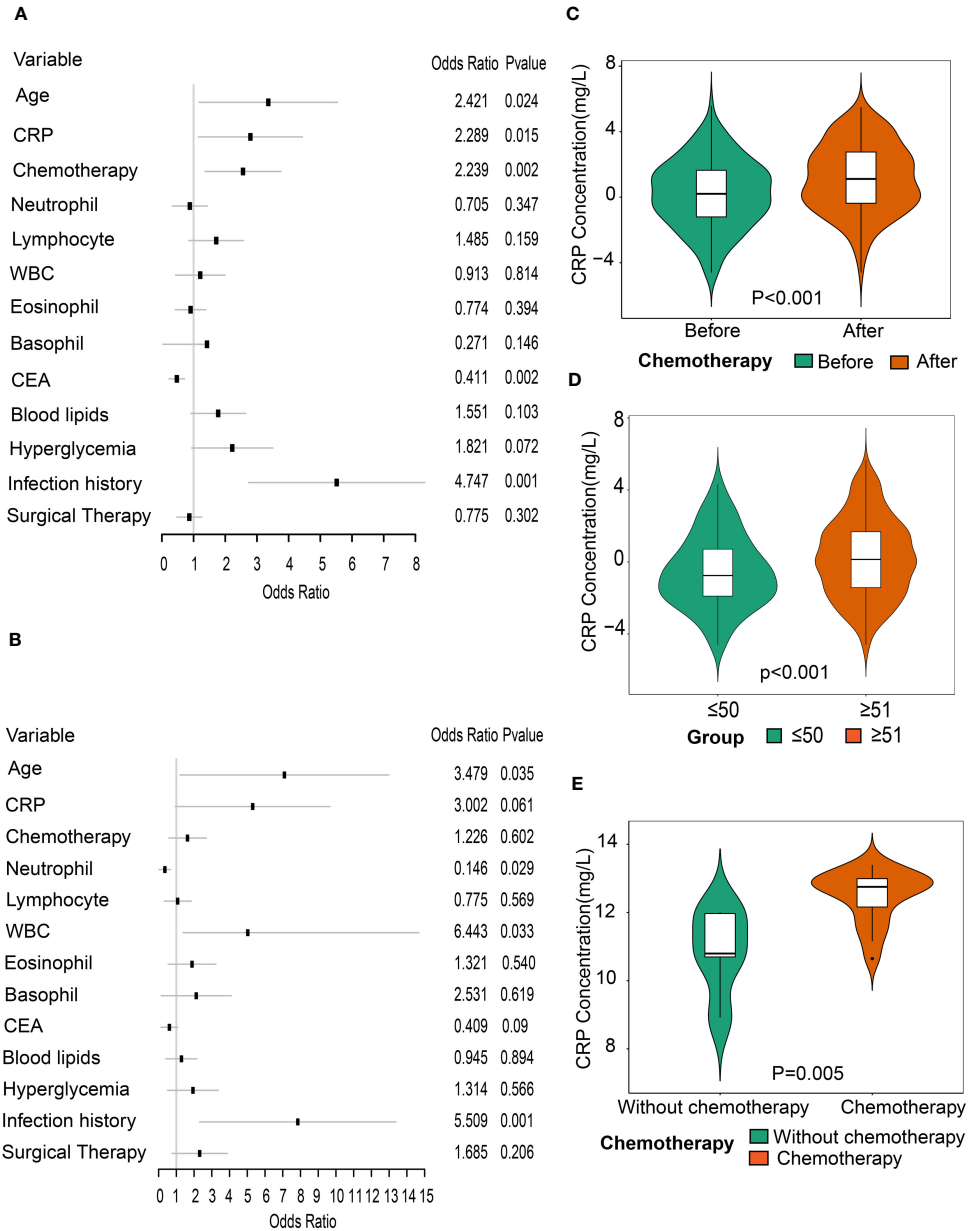
Association between CRP and CVD through subgroup analysis by gender. **(A)** Forest plot of all variables with odds ratios of CVD in male patients with rectal cancer. **(B)** Forest plot of all variables with odds ratios of CVD in female patients with rectal cancer. **(C)** Comparison of CRP levels between before and after chemotherapy in patients with rectal cancer (n=145). **(D)** Comparison of CRP levels between patients aged ≤50 years and those aged ≥51 years post-chemotherapy. **(E)**. Comparison of CRP levels between the non-chemotherapy group (n=5) and the chemotherapy group (n=19) in GSE32384 dataset.

In view of systemic differences between men and women (26, 27), subgroup analyses by gender were performed to determine a specific association of CRP with CVD. The results showed that CRP was a predictor of CVD risk in men, whereas no correlation between CRP and CVD was observed in women (**Figures 3A, B**). What’s more, an interaction between CRP and gender was found in the entire population (p for interaction = 0.05).

### 3.4 Effects of chemotherapy on CRP levels in patients with rectal cancer

To evaluate the influence of chemotherapy on CRP levels, we compared CRP levels of patients with rectal cancer before and after chemotherapy. There were 536 rectal cancer patients previously treated with chemotherapy, of whom the CRP levels of 145 were examined. We found a significant increase in CRP levels after chemotherapy compared to before chemotherapy (p<0.001) (**Figure 3C**). Considering the impact of age on tolerance in patients following chemotherapy, we also investigated the levels of CRP between groups of those aged ≥ 51 years and ≤ 50 years after chemotherapy treatment. Results showed that CRP levels were higher in patients aged over 51 years old compared to those aged under 50 years old, suggesting that elderly patients were more vulnerable to chemotherapy (**Figure 3D**).

### 3.5 The effect of chemotherapy on the level of CRP mRNA

**Figure 4:**
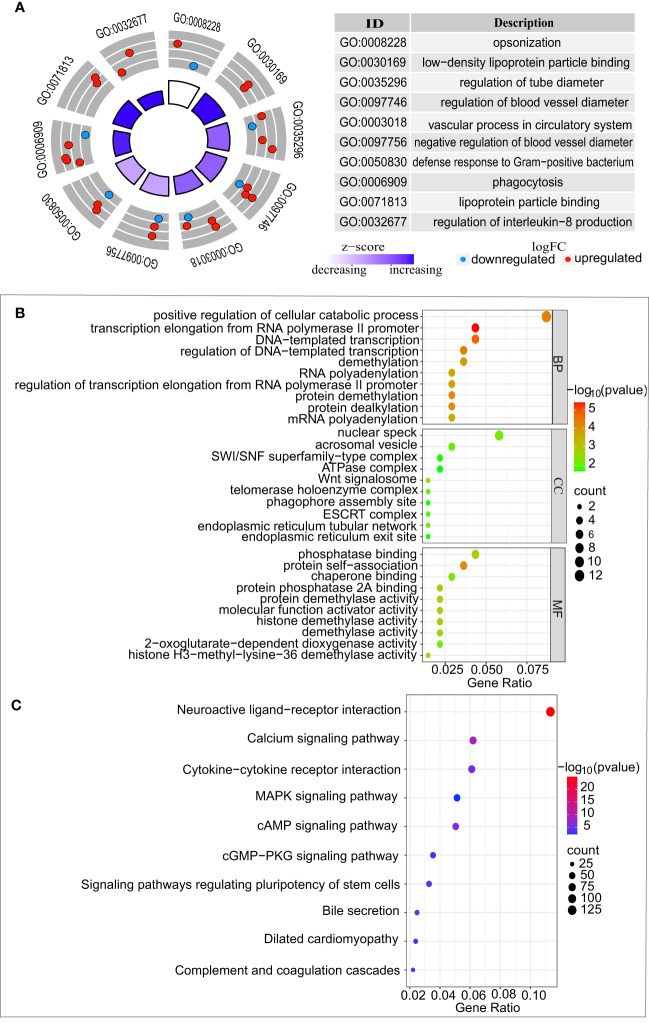
Functional enrichment analysis of differentially expressed genes between GSE32384 and GSE20060. **(A)** Biological pathway analysis of CRP enriched in colorectal liver metastases treated with chemotherapy from GSE32384. **(B)** GO analysis of genes displaying a positive association with CRP in vascular endothelium from GSE20060. **(C)** KEGG analysis of genes displaying a positive correlation with CRP in vascular endothelium from GSE20060.

The liver is not only the main release site for CRP but also the main pathway for chemotherapeutic metabolism. Accordingly, we selected the gene expression profile dataset of colorectal cancer liver metastases receiving oxaliplatin-based chemotherapy (GSE32384). To evaluate the impact of chemotherapy on CRP expression levels, we compared the differences in CRP levels between 19 samples with chemotherapy and five samples without any chemotherapy. The results showed that the CRP levels increased in the chemotherapy group in contrast to the group without any chemotherapy (p=0.005), indicating that chemotherapy can precipitate an increase in CRP (**Figure 3E**). Then, 96 eligible DEGs were identified by differential gene analysis between the two groups. To gain a better understanding of the role of differentially expressed genes in chemotherapy, we performed a GO analysis. The functional gene set analysis identified opsonization, low-density lipoprotein particle binding, regulation of tube diameter, regulation of blood vessel diameter vascular process in circulatory system, regulation of interleukin-8 production, etc. (**Figure 4A**).

### 3.6 The possible impact of CRP on the vascular endothelium

Oxidized phospholipids induce inflammatory responses via oxidative stress (28), which provides a possibility to explore the impact of CRP on vascular endothelium. We selected gene expression profile dataset of the vascular endothelial cells treated with oxidized phospholipids (GSE20060) simulating an inflammatory environment. To explore the possible biological functions and mechanisms of CRP in the vascular endothelium, we conducted DEG analyses, as well as GO and KEGG analyses.

In the GSE20060 dataset, a comparison between the oxidized and non-oxidized phospholipids treatments identified 2,696 DEGs. A Spearman correlation was conducted to analyze the CRP-related genes from the 2696 DEGs, which identified 140 genes. According to p < 0.05, the GO analysis of 140 CRP-related genes identified significantly enriched terms in biological processes (BP), cellular components (CC), and molecular functions (MF), as shown in (**Figure 4B**). Similarly, these CRP-related genes were significantly enriched in KEGG pathways, including neuroactive ligand-receptor interaction, calcium signaling pathway, Cytokine-cytokine receptor interaction, and so on (**Figure 4C**).

### 3.7 Identification of hub genes linking chemotherapy and CVD

We identified 30 common DEGs of the two datasets that were likely to be of great significance. To provide a broader understanding of the biological connections between chemotherapy and CVD, the existing functions of these genes were summarized in **Supplementary Table 1**. These current studies reported that 15 genes were associated with CVD, among which CCL1, LDLR, and TENT5A were identified as hub genes that had susceptibility effects for atherosclerosis (29–31). Our findings suggested that these hub genes were likely to be associated with genetic risk factors for atherosclerosis after chemotherapy.

## 4 Discussion

CRP is extensively used in clinical practice as a marker of inflammation, while the management of CRP and CVD in individuals with rectal cancer is frequently disregarded. To the best of our knowledge, this is the first attempt to systematically reveal the relationship between CRP and CVD among rectal cancer patients by combining macro and micro approaches, which may offer valuable scientific support for clinical treatment.

The present study identified the relationship between CRP and CVD in patients with rectal cancer. The results obviously indicated that CRP served as an independent risk factor for CVD in patients with rectal cancer after adjusting for potential confounding factors. Additionally, the ability of the model to predict CVD in patients with rectal cancer was improved by adding CRP, further highlighting the value of CRP in predicting CVD risk. The early American Heart Association and Centers for Disease Control and Prevention (AHA/CDC) have recommended that CRP levels of <1 mg/L, 1-3 mg/L, and >3 mg/L be used to assess the risk of CVD (32). A few studies demonstrated that elevated CRP levels above 3 mg/L could predict the occurrence of cardiovascular events and significantly increase the risk of all-cause and cardiovascular disease-related death (33, 34). Similar to these reports (33, 34), a high level (> 3.3 mg/L) of CRP was associated with an increased risk of CVD in rectal cancer patients. It was suggested that monitoring CRP levels might provide new clues and a means for assessing CVD risk in patients with rectal cancer. Despite the fact that the CRP has a predictive value for CVD in patients with rectal cancer, it is a nonspecific marker of inflammation and may be impacted by other conditions, such as bacterial or viral infections (35, 36), that have not been analyzed in our cohort. Consequently, a better understanding of the initial patient inflammation status may help to predict the risk of CVD.

We also demonstrated that chemotherapy had an impact on CRP levels, as evidenced by elevated levels of CRP in rectal cancer patients treated with chemotherapy. It seems possible that this result is due to a pro-inflammatory response caused by chemotherapy, which activates inflammation cytokines and increases their serum concentration (37). Transcriptional induction of the CRP gene in liver hepatocytes is proposed as a response to elevated levels of inflammatory cytokines (38). Moreover, chemotherapy-induced inflammation is also associated with aging. Cell senescence is a mechanism of aging that is exacerbated by chemotherapy and the accumulation of senescent cells results in the secretion of various pro-inflammatory factors, such as CRP (39, 40). As in our study, we identified that patients over 51 years old who were undergoing chemotherapy had higher CRP levels than those who were younger than 50 years old. Collectively, these works support the hypothesis that an increased CRP is the outcome of a treatment-induced inflammatory response.

Among men and women, there were significant dissimilarities in the relationship between CRP and CVD. A study by Isabella Kardys et al. (41) has shown that CRP was robustly and independently associated with the occurrence of heart failure in men, and the link was weaker among women. However, another study indicated that CRP was related to an increased risk of acute coronary syndrome in young women (42). In our study, subgroup analyses by gender in rectal cancer patients found an association between CRP levels and the risk of CVD only in men, whereas no such association was observed in women. A potential explanation for this result was that it was due to the anti-inflammatory effects exhibited by women compared to age-matched men (43).

Studies conducted at the molecular level further confirmed that chemotherapy-induced inflammation of the microenvironment could lead to an increase in CRP levels. The 96 significant DEGs showed enrichment of important functional pathways, in which CRP was involved in regulating IL-8 and the binding of low-density lipoprotein particles. Previous studies suggested that the pro-inflammatory effect of CRP was closely related to the induced secretion of the pro-inflammatory cytokine interleukin-8 (IL-8) and monocyte chemotactic protein 1 (MCP-1) (44). Atherosclerosis is the pathological basis of CVD. The binding of low-density lipoprotein (LDL) particles is essential in the development and progression of atherosclerotic cardiovascular disease (ASCVD) (45). CRP has the capability to bind to LDL particles through its phosphocholine binding site (46), which contributes to the formation of foam cells and the development of atherosclerosis. Hence, chemotherapy-induced increase in CRP levels may be one of the mechanisms that can lead to the development of CVD. Our study went one step further and explored the potential mechanism of CRP in the vascular endothelium. Our research demonstrated involvement of the Wnt signalosome pathway in CVD processes, which is in accordance with other studies (47–49). This highlights the role of the Wnt signalosome pathway in various heart and vascular diseases. Even though the Wnt signalosome pathway has limited activity in the cardiovascular system of healthy adults, its influence on CVD is still present. Furthermore, Marinou et al. (50) discussed the regulatory role of the Wnt signaling pathway in the formation and development of atherosclerosis, which gave rise to the potential for the development of effective medicines to combat CVD.

We also discovered that the neuroactive ligand-receptor interaction pathway was involved in the mechanisms of vascular disease, implying that CRP-related genes within this pathway could be responsible for the advancement of atherosclerosis via vascular endothelium. Indeed, recent research revealed that sympathetic nerve activation can cause malignant arrhythmias and dysfunction of the parasympathetic nervous system that can lead to heart failure (51). The neuroactive ligand-receptor interaction pathway was validated as potentially useful in the early diagnosis of acute coronary syndrome (ACS) (52) and arrhythmogenic right ventricular cardiomyopathy (53). Accordingly, neuroactive ligand-receptor interactions pathway may represent a novel mechanism for atherosclerosis, and more work is urgently needed to elucidate the mechanism. Besides, our study identified a significant enrichment of calcium pathways. M.J. Plank et al. have indicated that low levels of calcium in endothelial cells were a contributing factor to the high incidence of atherosclerosis in regions of disturbed blood flow (54).

We identified 30 genes, of which CCL1, LDLR, and TENT5A were found to be hub genes involved in the progression of atherosclerosis (29–31) and played an important role in the genetic risk of atherosclerosis. Simultaneously, 12 of the 30 genes had evidence of involvement in CVD by regulating signaling pathways. Moreover, the other 15 genes are still to be further determined for their role in the occurrence of CVD.

There were some limitations in our study. First, upon retrospective analysis, it was possible that there was a lack of standardization and incomplete recording in the diagnosis of cardiovascular events and the measurement of CRP level data, which could result in a research bias. Second, the cardiovascular risks beyond the scope of the electronic medical record system, such as lifestyles of exposure to out-of-hospital settings, were not accessible, meaning that potential confounders may not have been adequately controlled. Third, all the samples in this study were taken from a single hospital, the research results couldn’t be generalized to the wider geographical population. A further study with more focus on investigating this association in a larger population is needed. Finally, owing to the cross-sectional nature of this research, longitudinal studies are needed to examine associations over time, and prospective studies are recommended for the future.

## 5 Conclusion

In our research, we have found that CRP levels are linked to CVD in rectal cancer patients, and that chemotherapy-induced higher CRP levels are known to affect the vascular endothelium in cardiovascular disease. This could potentially offer a better understanding of the predictive and diagnostic value of CVD in rectal cancer patients in clinical practice.

## Data availability statement

The datasets presented in this study can be found in online repositories. The names of the repository/repositories and accession number(s) can be found below: https://www.ncbi.nlm.nih.gov/geo/, GSE32384 https://www.ncbi.nlm.nih.gov/geo/, GSE20060.

## Ethics statement

The studies involving humans were approved by research ethics committees of First Affiliated Hospital of Gannan Medical University. The studies were conducted in accordance with the local legislation and institutional requirements. Written informed consent for participation was not required from the participants or the participants’ legal guardians/next of kin in accordance with the national legislation and institutional requirements.

## Author contributions

HQ: Methodology, Software, Visualization, Writing – original draft, Writing – review & editing. CW: Resources, Supervision, Writing – review & editing. CY: Data curation, Supervision, Writing – review & editing. LL: Data curation, Resources, Supervision, Writing – review & editing. YC: Supervision, Writing – review & editing. YL: Supervision, Writing – review & editing, Funding acquisition. XZ: Data curation, Supervision, Writing – review & editing. YG: Conceptualization, Funding acquisition, Project administration, Supervision, Writing – review & editing.
